# Camera–LiDAR Wide Range Calibration in Traffic Surveillance Systems

**DOI:** 10.3390/s25030974

**Published:** 2025-02-06

**Authors:** Byung-Jin Jang, Taek-Lim Kim, Tae-Hyoung Park

**Affiliations:** 1 Department of Intelligent Systems and Robotics, Chungbuk National University, Cheongju 28644, Republic of Korea; jbj1029jbj@chungbuk.ac.kr; 2 Department of Control and Robot Engineering, Chungbuk National University, Cheongju 28644, Republic of Korea; taeglem@chungbuk.ac.kr

**Keywords:** calibration, optimization, genetic algorithm, sensors, infrastructure

## Abstract

In traffic surveillance systems, accurate camera–LiDAR calibration is critical for effective detection and robust environmental recognition. Due to the significant distances at which sensors are positioned to cover extensive areas and minimize blind spots, the calibration search space expands, increasing the complexity of the optimization process. This study proposes a novel target-less calibration method that leverages dynamic objects, specifically, moving vehicles, to constrain the calibration search range and enhance accuracy. To address the challenges of the expanded search space, we employ a genetic algorithm-based optimization technique, which reduces the risk of converging to local optima. Experimental results on both the TUM public dataset and our proprietary dataset indicate that the proposed method achieves high calibration accuracy, which is particularly suitable for traffic surveillance applications requiring wide-area calibration. This approach holds promise for enhancing sensor fusion accuracy in complex surveillance environments.

## 1. Introduction

**Figure 1:**
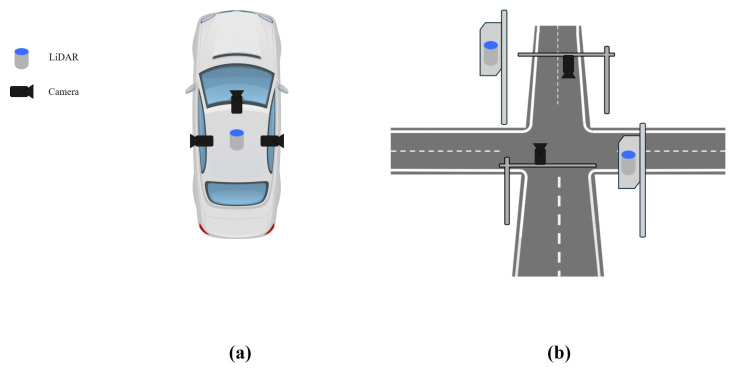
(**a**) Example of camera and LiDAR sensor placement on a vehicle, (**b**) Example of camera and LiDAR sensor placement at an intersection.

A traffic surveillance system [1,2] delivers robust performance in monitoring traffic flow and ensuring the safety of drivers and pedestrians. In these systems, cameras and LiDAR act as recognition sensors [3], accurately recognizing objects by interpreting rich textures in camera images using neural networks. LiDAR, on the other hand, emits laser beams and measures the intensity and time of reflected light, providing precise information about distance and reflectivity. To effectively detect and measure unexpected incidents, such as accidents or jaywalking [4,5], traffic surveillance systems [6] require calibration. In autonomous vehicles, as illustrated in Figure 1a, the calibration of extrinsic parameters is conducted within a relatively narrow range, focusing on the precise alignment of onboard sensors. However, in a traffic surveillance system, as shown in Figure 1b, the calibration must cover a broader range, taking into account multiple sensors distributed over a wide intersection area to ensure comprehensive monitoring.

Existing calibration studies can be categorized into target-based and target-less methods [7,8]. In the target-based method, the coordinate systems of the two sensors are unified with high accuracy using artificial landmarks. Although the target-based method provides high accuracy at short distances, achieving accurate calibration at long distances is challenging due to difficulties in recognizing artificial landmarks. Therefore, the target-less method is more suitable for traffic surveillance. Levinson et al. [9] performed calibration using LiDAR discontinuities and image object edges for online calibration in a road environment. Levinson’s method is an edge-based calibration approach that has proven its performance across various datasets. While it is effective for precise calibration over a small area by globally extracting features, it often gets stuck in local optima when applied to a broader range.

Recent deep learning studies [10,11] have demonstrated high performance across various applications. In calibration research, segmentation networks [12,13] have been utilized to calibrate camera–LiDAR systems by matching segmented object edges with LiDAR discontinuities, achieving high precision. However, while segmentation methods perform well in familiar environments, they struggle with unfamiliar settings or new datasets. To address this issue, Luo et al. [14] proposed a method that achieves high-precision calibration without prior training, focusing on a localized range. Despite its effectiveness, this method is limited when applied to broader areas. Therefore, we propose a genetic algorithm (GA)-based camera–LiDAR calibration approach for wide-area traffic surveillance systems.

In wide-area traffic surveillance, ensuring accurate calibration is essential for effectively integrating sensor data. It is crucial to extract common features over a wide range for camera–LiDAR calibration in traffic surveillance system. Existing methods have used edge and depth discontinuities to extract common features, but more specific candidates are needed for wide-range calibration. To achieve this, we reduced the search space by detecting dynamic objects and extracting features from both sensors. In our approach, we represented the calibration parameters as chromosomes in a genetic algorithm and used surrounding candidates to find the global optimum as the objective function.

The contributions of this paper are as follows.

- To explore camera–LIDAR calibration parameters in a wide range of search areas, we combined dynamic objects and the GA to achieve efficient calibration parameter exploration that does not require learning.
- We efficiently performed dynamic object detection using non-deep learning-based algorithms and sensor fusion methods to achieve robust performance even when the evaluation environment changes.
- We designed the genetic algorithm to find a global rather than a local optimum by optimizing the objective function and the crossover and mutation processes, which are the reproduction processes.

## 2. Related Work

Camera and LiDAR measure the surrounding environment differently, each using its coordinate system axes. Calculating the transformation between different coordinate systems is essential for effectively fusing data, a process known as extrinsic parameter estimation. Existing studies on extrinsic parameter estimation can be broadly classified into three methods:

- Target methods, which use artificial landmarks like checkerboards or artificial markers for calibration;
- Target-less methods, which rely on extracting natural features from the surrounding environment for matching;
- Deep learning-based methods, which leverage data-driven models to estimate relationships, such as end-to-end segmentation networks between coordinate systems.

Each method has strengths, depending on the accuracy requirements, ease of setup, and environmental conditions.

### 2.1. Target-Based Method

Target-based methods using artificial landmarks have the advantage of robustly responding to various environmental changes according to the geometric information of the artificial landmarks. They provide high precision and can reduce the algorithm’s complexity by clearly extracting features with geometric information known in advance. The checkerboard [7] can be measured from intrinsic or extrinsic calibration parameters. Checkerboard [15] is a square grid pattern with features that can be converted into a problem of extracting areas through corners where feature points intersect and finding common areas with lidar. When a sphere is used as an artificial landmark, it has the advantage of being able to perform calibration regardless of the viewpoint [16]. Using the features of circles, Carlos Guindel et al. [17] extracted four circles on a plane and performed matching by correcting translation through center point matching and assuming no significant change in rotation. Triangles [18] allow for robust calibration for various viewpoints and accurate calibration; it is crucial to know the shape and size of the geometric information [19]. Using a specific size box makes it possible to estimate the position at the corner point and makes it easier to find the coordinate system for the camera and LiDAR. Markers with patterns such as Aruco markers estimate the axis of the artificial landmark as a recognition result and perform sensor calibration [20].

The target method based on such artificial landmarks estimates the extrinsic parameters of the camera and LiDAR by utilizing geometry information, which is relatively robust and accurate compared to other methods. However, in the case of installing artificial landmarks on autonomous vehicles or road traffic surveillance systems, real-time calibration is required, but the artificial landmark-based method presents difficulties.

### 2.2. Target-Less Method

Unlike target-based approaches, target-less methods extract features directly from the natural environment instead of relying on artificial landmarks. The target-less method primarily uses geometric features, such as edges [9], and performs calibration by leveraging various edges and LiDAR discontinuities in the image. Since natural landmarks are used instead of artificial ones, many features are extracted, which increases the risk of converging to a local optimum. To address this, the inverse distance transform was applied. Yuan et al. [8] analyzed features of discontinuous in-depth edges, such as foreground and background, with LiDAR to efficiently utilize indoor environments and high-density data. MFCalib [21], which utilizes depth discontinuities and depth continuous edges, performed iterative optimization to reduce calibration errors further. There is also a method that improves computational efficiency and performance by extracting lines [22,23] from edges, but it is difficult to find external parameters when features are extracted in a tiny area. Previous studies [24,25] address the challenge of finding an optimal solution, and the optimization problem is particularly difficult in calibration tasks that involve extracting numerous features.

Recently, in robotics, there have been studies on finding extrinsic parameters from the perspective of motion rather than geometric features in the fields of autonomous vehicles and mobile robots [26]. The time acquired from the sensor is different due to the motion, and this calibration requires not only spatial but also temporal calibration, which corresponds to synchronization [27,28,29]. A study on synchronizing sensors by expressing the parallax due to motion as the path of a moving object [29] and correcting the path is one of the studies suitable for the road traffic environment attached to the roadside. A study on compensating for sensor shake in a moving situation using the IMU sensor [30] has a high potential for practical use but is still efficient in a small search space.

### 2.3. Deep Learning Method

Deep learning-based methods either utilize networks specifically for semantic feature extraction or perform end-to-end calibration by combining feature extraction and parameter regression.

Calibration accuracy can be improved by matching geometric features with semantic information using a semantic segmentation network. Zhu et al. [31] introduced an online calibration method that aligns object regions between sensors using semantic information. Liao et al. [32] developed SE-Calib, which matches object semantic edges in urban environments, and Liu et al. [33] proposed SemAlign, using a semantic alignment loss to perform calibration without annotated data. Kodaira et al. [34] extended these approaches by introducing network-based feature extraction to calibrate both spatiotemporal parameters, while Luo et al. [14] proposed a zero-training method based on the Segment Anything Model, aiming to eliminate dependence on labeled data.

RegNet [35], which first applied the end-to-end approach to calibration, integrates feature extraction, matching, and regression within a single CNN to enable real-time calibration. CalibNet [36] employs a 3D spatial transformer layer to align camera images and point clouds using photometric and point cloud distance losses. LCCNet [37] calculates a cost volume for feature matching between RGB images and LiDAR data to estimate transformations. CalibRCNN [38] combines CNN and LSTM layers to capture temporal information across frames, reducing noise impacts in calibration. RGGNet [39] introduces a tolerance-based loss function, leveraging Riemannian geometry and deep generative models to maintain stable relative alignment.

Deep learning-based methods have the limitation of relying on trained environments. In untrained environments or when data conditions change, their performance can degrade significantly.

## 3. Notation and Problem Definition

This study assumes that the data collected from the camera and LiDAR sensors are time-synchronized. Table 1 is the mathematical representation of the entire paper, which is explained in this section and in the proposed method. Camera–LiDAR calibration refers to the process of estimating the transformation parameters that define the spatial relationship between the LiDAR and camera sensors.

One of the most traditional methods for camera–LiDAR calibration utilizes a checkerboard pattern, as described by Zhang et al. [7]. The calibration problem is formulated as a least squares optimization task that seeks to minimize the distance between the projected 3D LiDAR points and the corresponding 2D image pixels. This minimization is achieved through the 3D-to-2D projection process outlined below. Various optimization techniques have been explored to solve this minimization problem, making the calibration task a quintessential example of an optimization challenge.

The LiDAR point cloud is denoted as $P^{L}\in R^{3}$, where *L* represents the LiDAR coordinate. Each point in the point cloud $P^{L}=\left\{p_{1}^{L},p_{2}^{L},p_{3}^{L},\cdots ,p_{i}^{L},\cdots ,p_{n}^{L}\right\}$ is represented as $p_{i}^{L}\in R^{3}$, consisting of $\left(x_{i}^{L},y_{i}^{L},z_{i}^{L}\right)$. Here, *n* denotes the total number of points in the current point cloud frame. The transformation relationship between the LiDAR coordinate and the camera coordinate is expressed in Equation (1):

(1) $$\left[\begin{array}{c} x_{i}^{C}\\ y_{i}^{C}\\ z_{i}^{C}\\ 1 \end{array}\right]=\left[\begin{array}{cc} R & T\\ 0^{⊤} & 1 \end{array}\right]\left[\begin{array}{c} x_{i}^{L}\\ y_{i}^{L}\\ z_{i}^{L}\\ 1 \end{array}\right]$$

where R is the 3×3 rotation matrix in Equation (2):

(2) $$R=\left[\begin{array}{ccc} r_{11} & r_{12} & r_{13}\\ r_{21} & r_{22} & r_{23}\\ r_{31} & r_{32} & r_{33} \end{array}\right]$$

and T is the 3×1 translation vector in Equation (3):

(3) $$T=\left[\begin{array}{c} t_{x}\\ t_{y}\\ t_{z} \end{array}\right]$$

The coordinate transformation matrix, composed of the rotational matrix R and the translational vector T, is represented as a 3×4 matrix with 6 degrees of freedom (6-DOF), which is defined as the extrinsic parameters.

The point cloud transformed from the LiDAR coordinate to the camera coordinate is denoted as $P^{C}\in R^{3n}$, where *C* indicates the camera coordinate system. Each point in the camera coordinate system point cloud $P^{C}=\left\{p_{1}^{C},p_{2}^{C},p_{3}^{C},\cdots ,p_{i}^{C},\cdots ,p_{n}^{C}\right\}$ is represented as $\left(x_{i}^{C},y_{i}^{C},z_{i}^{C}\right)$. Each point $p_{i}^{C}=\left(x_{i}^{C},y_{i}^{C},z_{i}^{C}\right)$ transformed to the camera coordinate system is projected onto the image plane coordinate using Equation (4):

To ensure accurate projection, the normalization of intrinsic parameters must be explicitly included. The projection equation is

(4) $$\left[\begin{array}{c} u\\ v\\ 1 \end{array}\right]=\frac{1}{z_{i}^{C}}K\left[\begin{array}{c} x_{i}^{C}\\ y_{i}^{C}\\ z_{i}^{C} \end{array}\right]$$

where K is the 3×3 intrinsic matrix, defined as

(5) $$K=\left[\begin{array}{ccc} f_{x} & 0 & c_{x}\\ 0 & f_{y} & c_{y}\\ 0 & 0 & 1 \end{array}\right].$$

Here, $f_{x},f_{y}$ are the focal lengths, and $c_{x},c_{y}$ represent the optical center coordinates.

The set of pixels projected onto the image plane coordinate is denoted as $P^{I}=\left\{p_{1}^{I},p_{2}^{I},p_{3}^{I},\cdots ,p_{i}^{I},\cdots ,p_{n}^{I}\right\}$, where *I* represents the image plane coordinate, and each pixel is represented as $p_{i}^{I}=\left(u_{i},v_{i}\right)\in Z^{2}$. The combined coordinate transformation relationship from Equations (1) and (4) is expressed in Equation (6):

(6) $$\left[\begin{array}{c} u_{i}\\ v_{i}\\ 1 \end{array}\right]=\frac{1}{z_{i}^{C}}K\left[\begin{array}{cc} R & T\\ 0^{⊤} & 1 \end{array}\right]\left[\begin{array}{c} x_{i}^{L}\\ y_{i}^{L}\\ z_{i}^{L}\\ 1 \end{array}\right]$$

LiDAR point cloud features are projected onto the image plane using Equation (6). By maximizing the alignment between the features extracted from the camera image and the projected LiDAR features, this study estimates the precise extrinsic parameters $\left(R^{*},T^{*}\right)$. These parameters define the coordinate transformation between the two sensors, achieving precise alignment through an optimization process.

## 4. Proposed Method

**Figure 2:**
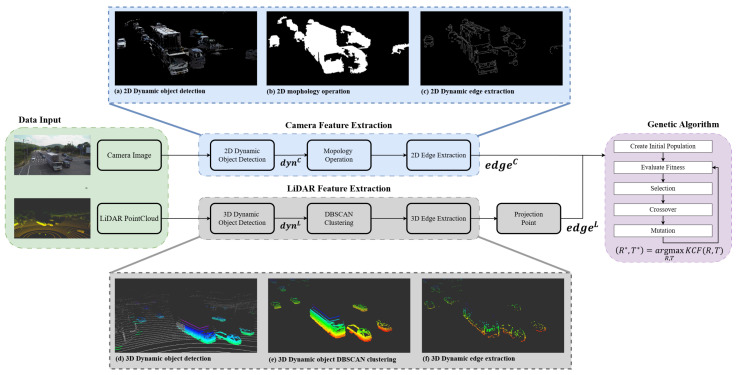
Proposed calibration system configuration diagram and feature extraction results.

The proposed method comprises three main components: LiDAR feature extraction, camera feature extraction, and optimization using a genetic algorithm. The overall system architecture is illustrated in Figure 2.

In road traffic surveillance applications, the physical distance between the camera and LiDAR sensors is typically substantial. This increased separation results in a broader search space for calibration parameters, making the calibration process more challenging. Consequently, identifying a global optimum becomes essential to avoid convergence to local optima, which can significantly degrade calibration accuracy. Traditional local search methods often struggle in such extensive search spaces due to their propensity to become trapped in suboptimal solutions. In contrast, the genetic algorithm excels in exploring wide and complex search spaces efficiently. Its inherent mechanisms, such as selection, crossover, and mutation, enable the algorithm to maintain diversity within the population of solutions, thereby reducing the likelihood of premature convergence and enhancing the chances of discovering the global optimum.

Furthermore, leveraging edge features of dynamic objects for calibration offers a strategic advantage in minimizing unnecessary edge noise from static backgrounds. Dynamic objects, such as moving vehicles, are prevalent in road environments and provide distinct and easily identifiable features. By focusing on these dynamic edges, the method effectively filters out irrelevant static background information, which can otherwise introduce noise and reduce calibration precision. The utilization of dynamic objects ensures that the extracted features are both prominent and reliable, facilitating more accurate alignment between the camera and LiDAR data. This approach not only enhances the robustness of the calibration process but also improves its adaptability to varying environmental conditions commonly encountered in traffic surveillance scenarios.

In summary, the integration of LiDAR and camera feature extraction with a genetic algorithm-based optimization framework addresses the challenges posed by large search spaces and noisy static backgrounds. This comprehensive methodology ensures high calibration accuracy and reliability, making it well-suited for demanding applications in road traffic surveillance.

### 4.1. LiDAR Feature Extraction

**Figure 3:**
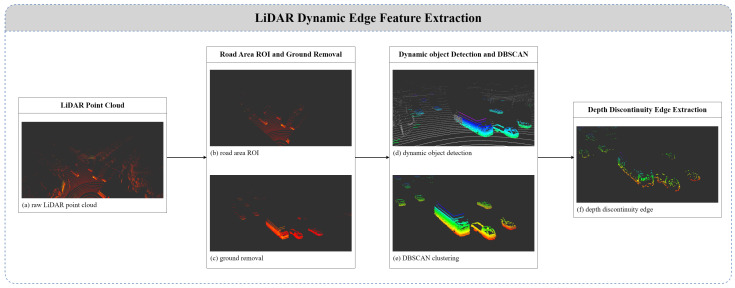
Flowchart of LiDAR feature extraction.

The LiDAR feature extraction process involves detecting 3D dynamic objects, followed by applying clustering for noise reduction, as illustrated in the Figure 3. Subsequently, edge features are extracted based on depth discontinuities. To enhance the processing efficiency of LiDAR point clouds, only the road region is selected as the region of interest (ROI), and a ground removal algorithm is applied to extract objects located on the road. Figure 3c shows the result of performing the ground removal algorithm. To detect dynamic objects, a point cloud buffer is used to store *F* frames. The LiDAR point cloud $P_{f}^{L}$ collected at each frame *f* consists of *n* points. Each point $p_{f,i}^{L}\in R^{3}$ is represented by the 3D coordinates $\left(x_{f,i}^{L},y_{f,i}^{L},z_{f,i}^{L}\right)$. A kd-tree is used to find the nearest neighbor point $p_{f-1,i}^{L}$ in the previous frame $p_{f-1}^{L}$, and the Euclidean distance ${\mathrm{dist}}_{f,i}$ between the current and the previous frame is calculated using Equation (7).

(7) $${\mathrm{dist}}_{f,i}^{L}={∥p_{f,i}^{L}-p_{f-1,i}^{L}∥}^{2}$$

Subsequently, the average distance ${\bar{dist}}_{i}^{L}$ of the distance differences obtained within the point cloud buffer is computed over *F* frames for each point, as shown in Equation (8).

(8) $${\bar{\mathrm{dist}}}_{i}^{L}=\frac{1}{F}\sum_{k=1}^{F}{\mathrm{dist}}_{f-k,i}^{L}$$

Points with an average distance ${\bar{dist}}_{i}^{L}$ exceeding a predefined threshold are classified as dynamic using Equation (9). This method effectively distinguishes between the stationary point cloud and dynamic point cloud ${\mathrm{dyn}}^{L}$.

(9) $$\mathrm{if}\;{\bar{dist}}_{i}^{L}>th_{dynamic}^{L},\;\mathrm{then}\;p_{i}^{L}\in {\mathrm{dyn}}^{L}$$

Figure 3d is used to indicate dynamic objects. Although ground removal was applied in Figure 3c, the static background points are displayed in white to distinguish them from the dynamic points. The dynamic object result requires segmentation work for each object, as shown in Figure 3d. LiDAR point clouds often exhibit regions with sparse noise, necessitating the extraction of high-density object clusters. To address this, we employd the DBSCAN (density-based spatial clustering of applications with noise) [40] algorithm, which effectively clusters points representing objects and discards low-density noise outliers. After dynamic object detection and clustering, depth discontinuities D are calculated using Equation (10).

(10) $$D=\max(∥p_{i-1}^{L}-p_{i}^{L}∥,∥p_{i+1}^{L}-p_{i}^{L}∥,0)$$

Depth discontinuities are calculated from distance differences between neighboring points. Points with high discontinuities are extracted as LiDAR edge point clouds, representing object boundaries on the road.

To handle distant objects where edges appear faint, we define an adaptive threshold $th_{depth}^{\prime }\left(k\right)$ that decreases with distance. Let $d_{k}=∥C_{k}^{L}∥$ be the distance from the LiDAR origin to the centroid of cluster *k*. Then, $th_{depth}^{\prime }\left(k\right)$ is given by

(11) $$th_{depth}^{\prime }\left(k\right)=th_{depth}\times g\left(d_{k}\right),$$

where $th_{depth}$ is a base threshold, and g(·) is a decreasing function of distance. For example,

(12) $$g\left(d_{k}\right)=\frac{1}{1+\alpha \;d_{k}},$$

with α>0. As $d_{k}$ increases, $g(d_{k})$ decreases, making $th_{depth}^{\prime }\left(k\right)$ smaller for distant objects. Replacing $th_{depth}$ with $th_{depth}^{\prime }\left(k\right)$ in Equation (11) helps maintain robust edge detection over varying distances:

(13) $$\mathrm{if}\;D>th_{depth}^{\prime }\left(k\right),\;\mathrm{then}\;p_{i}^{L}\in {\mathrm{edge}}^{L}.$$

### 4.2. Camera Feature Extraction

**Figure 4:**
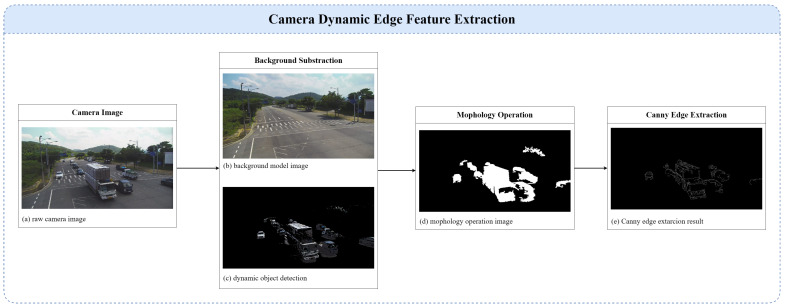
Flowchart of camera feature extraction: background substraction, dynamic object detection, mophology operation, and canny edge extraction.

Camera feature extraction comprises 2D dynamic object detection, morphological operations for noise removal, and Canny edge extraction processes, as illustrated in Figure 4. For fixed-position cameras, dynamic objects can be effectively detected using a median approximation background subtraction algorithm [41]. In this approach, background modeling is performed by incrementally updating the background model $I{\left(u,v\right)}^{\mathrm{background}}$ with a median filter applied over a sequence of previous frames. As shown in Figure 4b, when a new frame is captured, each pixel intensity in the current frame $I{\left(u,v\right)}^{\mathrm{current}}$ is compared with the background model. Pixels with an intensity difference greater than a predefined threshold $th_{\mathrm{dynamic}}^{C}$ are classified as dynamic objects ${\mathrm{dyn}}^{C}$.

(14) $$\Delta I\left(u,v\right)=\left|I{\left(u,v\right)}^{\mathrm{background}}-I{\left(u,v\right)}^{\mathrm{current}}\right|$$

(15) $$\mathrm{if}\;\Delta I\left(u,v\right)>th_{\mathrm{dynamic}}^{C},\;\mathrm{then}\;I{\left(u,v\right)}^{\mathrm{current}}\in {\mathrm{dyn}}^{C}$$

In Figure 4, binarization is performed in the process from Figure 4c to Figure 4d to separate foreground objects from the background. This method ensures that the background model adapts over time, enabling robust detection of dynamic objects in fixed environments, even under varying lighting and environmental conditions.

Morphological operation is applied to the detected foreground objects to remove noise and correct object shapes in Figure 4d. Initially, erosion is applied to eliminate small noise by refining the object’s boundaries. Next, dilation is applied to expand boundaries, restoring its original shape and making boundaries clearer. By combining erosion and dilation operations into an opening operation, object shapes are corrected, and the influence of small noise is minimized.

After applying morphological operations, we set the resulting object mask as the region of interest and then apply Canny edge detection to extract object boundaries. Canny edge detection is effective at highlighting areas with abrupt intensity changes, but smaller objects can be more susceptible to noise or weak gradients. To address this, we define the lower and upper Canny hysteresis thresholds as functions of the mask area $A_{k}$ for the *k*-th object:

(16) $$\begin{array}{cc} th_{low}^{\prime }\left(A_{k}\right) & =th_{low}+\alpha \;A_{k},\\ th_{high}^{\prime }\left(A_{k}\right) & =th_{high}+\beta \;A_{k}, \end{array}$$

where $A_{k}$ is the size of the mask in pixels, $th_{low}$ and $th_{high}$ are the base thresholds satisfying $0\leq th_{low}<th_{high}$, and α,β>0 are scaling factors controlling how quickly the thresholds increase with the mask area. For larger areas, $th_{low}^{\prime }$ and $th_{high}^{\prime }$ become higher, preventing excessive edge detection. For smaller areas, they remain lower to capture weaker edges more reliably. By substituting $th_{low}^{\prime }\left(A_{k}\right)$ and $th_{high}^{\prime }\left(A_{k}\right)$ into the Canny algorithm’s hysteresis thresholding step, we achieve consistent edge detection across varying object sizes.

### 4.3. Calibration Using Real-Coded Genetic Algorithm

For the camera–LiDAR calibration, a real-coded genetic algorithm [42] was applied. Unlike a binary-coded genetic algorithm, the real-coded genetic algorithm represents genes as real numbers, which allows for higher precision in continuous search spaces. The camera–LiDAR calibration problem involves estimating extrinsic parameters such as roll,pitch,yaw, $t_{x},t_{y},$ and $t_{z}$, which are continuous values in coordinate transformation. Therefore, the real-coded genetic algorithm is particularly suitable for precisely searching through this continuous parameter space.

#### 4.3.1. Overview of Genetic Algorithm

**Figure 5:**
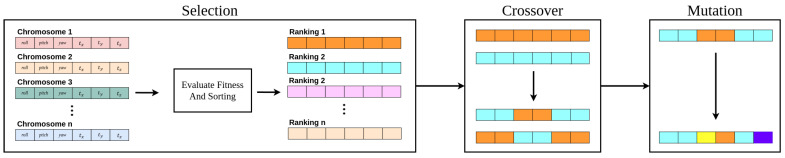
Regeneration process of genetic algorithm.

The genetic algorithm is an optimization technique inspired by the principles of natural evolution, designed to identify optimal solutions for complex problems. It iteratively improves solutions through a process of evolution, utilizing three primary operations: selection, crossover, and mutation. Each candidate solution is encoded as a chromosome, as depicted in Figure 5, with six genes representing the coordinate transformation parameters between the camera and LiDAR: rotation angles roll,pitch,yaw, and translation parameters $t_{x},t_{y},$ and $t_{z}$. The chromosome of the *i*-th individual is defined as ${ch}_{i}=\left\{{roll}_{i},{pitch}_{i},{yaw}_{i},t_{x,i},t_{y,i},t_{z,i}\right\}\in R^{6}$.

#### 4.3.2. Fitness Function

In Equation (17), *K* is the intrinsic parameter of the camera, *R* is the rotation matrix, and *T* is the translation vector. Using this equation, LiDAR edge points $edge_{i}^{L}\in R^{3}$ are projected onto the image plane as $p_{i}^{I}=\mathrm{Proj}\left(R,T,edge_{i}^{L}\right)\in Z^{2}$.

(17) $$\mathrm{Proj}\left(R,T,p\right)=K\cdot \left(R\cdot p+T\right),$$

(18) $$I^{kNN}=\mathrm{kNN}\left(p_{i}^{I},{\mathrm{edge}}^{C},k\right),$$

As shown in Equation (18), a k-nearest neighbor (kNN) search is used to find the set of *k* nearest camera edge pixels $I^{kNN}=\left\{\;edge_{1}^{C},edge_{2}^{C},\cdots ,edge_{k}^{C}\right\}$ from the full set of camera edge pixels ${\mathrm{edge}}^{C}$ for each projected LiDAR edge point $p_{i}^{I}$. Each $edge_{j}^{C}\in Z^{2}$ lies in the image plane coordinate system.

(19) $$\mathrm{KCF}\left(R,T\right)=\sum_{p_{i}^{I}\;\in \;edge^{L}}\;\sum_{\;edge_{j}^{C}\;\in \;I^{kNN}}\exp\;\left(\;-\;\frac{{∥p_{i}^{I}-edge_{j}^{C}∥}^{2}}{2\;\lambda^{2}}\right).$$

Equation (19) measures the similarity between the projected LiDAR edge points and the corresponding camera edge pixels. For each projected point $p_{i}^{I}$, only the *k* nearest camera edge pixels, $I^{kNN}$ are included in the summation. This approach suppresses mismatches with distant edges and focuses on the most relevant neighbors. The distance $∥p_{i}^{I}-edge_{j}^{C}∥$ is converted to a correlation score via a Gaussian kernel [43], where λ controls the sensitivity to spatial discrepancies. A larger λ reduces the sensitivity to distance errors, while a smaller λ increases it. Ultimately, this kernel correlation function (KCF) score serves as the fitness function for each chromosome ${\mathrm{ch}}_{i}$, guiding the genetic algorithm toward optimal extrinsic parameters *R* and *T*.

Due to the large physical distance between the camera and LiDAR, significant viewpoint differences arise, leading to edge points that do not have exact correspondences between the two sensors. KCF effectively mitigates this issue by prioritizing overall structural alignment rather than enforcing direct point-to-point matching. The kernel function assigns higher weights to spatially close edges while minimizing the influence of misaligned or outlier points. As a result, the objective function remains smooth and differentiable, reducing the sensitivity to local minima even when edges do not perfectly match.

#### 4.3.3. Operations of the Real-Coded Genetic Algorithm

The genetic algorithm consists of three main stages: selection, crossover, and mutation. The optimization process follows the steps described in Algorithm 1 and aims to maximize the objective function defined in Equation (20).

(20) $$\left(R^{*},T^{*}\right)=\arg\max_{R,T}KCF\left(R,T\right)$$

In the first selection stage, ranking selection [44] is applied, prioritizing chromosomes with higher fitness scores. The selected chromosomes are then passed to the crossover stage. The probability of selection is calculated using Equation (21).

(21) $$Prob\left(rank\right)=\frac{1}{n}\left(sp-\left(2sp-2\right)\frac{rank-1}{n-1}\right),\;1\leq rank\leq n,\;1\leq sp\leq 2$$

Here, Prob(rank) represents the selection probability for a ranked chromosome, and sp is the selection pressure. When selection pressure is lowered, the probability that lower-ranked individuals are selected increases, thereby maintaining diversity. *n* is the total number of chromosomes.

(22) $$c_{i}=\alpha_{g}\cdot q_{1}+\left(1-\alpha_{g}\right)\cdot q_{2}$$

During the crossover stage, a blend crossover [45] is used to linearly mix genes from two parent individuals to create offspring. In Equation (22), $c_{i}$ represents the offspring, $q_{1}$ and $q_{2}$ are the parent individuals, and $\alpha_{g}\in \left[0,1\right]$ is a parameter that controls the range of blending.
**Algorithm 1** Optimization process of camera–LiDAR calibration.**Input**: Image edge pixels ${\mathrm{edge}}^{C}$, LiDAR edge points ${\mathrm{edge}}^{L}$,    Crossover probability $p_{c}$, Mutation probability $p_{m}$**Output**: Optimized extrinsic parameters ($R^{*},T^{*}$)1Initialize population using Equation (19)2**for** g=1 to $G_{max}$ **do**3  Compute selection probabilities using Equation (21)4  Select chromosomes based on probabilities (e.g., ranking selection)5  **for** each parent pair $\left(q_{1},q_{2}\right)$ **do**6    **if** $\mathrm{rand}()<p_{c}$ **then**7      Generate offspring: $c_{i}=\alpha \cdot q_{1}+\left(1-\alpha \right)\cdot q_{2}$(Equation (22))8      Update α: $\alpha \left(g\right)=\alpha_{0}\cdot \left(1-g/G_{max}\right)$(Equation (23))9    **else**10      Offspring is copied from one of the parents (e.g., $c_{i}\leftarrow q_{1}$)11    **end if**12  **end for**13  **for** each gene in offspring **do**14    **if** $\mathrm{rand}()<p_{m}$ **then**15      Mutate gene: ${\mathrm{gene}}_{\mathrm{mutated}}=\mathrm{gene}+N\left(0,\sigma_{g}^{2}\right)$(Equation (24))16      Update σ: $\sigma_{g}=\sigma_{0}\cdot \left(1-g/G_{max}\right)$(Equation (25))17    **else**18      No mutation is applied19    **end if**20  **end for**21  Evaluate fitness using Equation (19) and update population22  **if** termination condition is met (e.g., fitness threshold or $g=G_{max}$) **then**23    **break**24  **end if**25**end for**26**Return** Optimized extrinsic parameters ($R^{*},T^{*}$)

(23) $$\alpha_{g}=\alpha_{0}\cdot \left(1-\frac{g}{G_{max}}\right)$$

The adaptive value of α is defined as in Equation (23). $\alpha_{0}$ is the initial value of α, *g* is the current generation, and $G_{max}$ is the maximum number of generations. As the generations progress, the value of α decreases, enabling more fine-grained exploration.

(24) $${gene}_{mutated}=gene+N\left(0,\sigma_{g}^{2}\right)$$

(25) $$\sigma_{g}=\sigma_{0}\cdot \left(1-\frac{g}{G_{max}}\right)$$

In the mutation stage, a larger mutation rate is applied to genes in the early generations to explore a broader search space, while the mutation rate is reduced in later generations for more precise search. The mutation process is defined by Equation (24). $N(0,\sigma_{g}^{2})$ represents a value sampled from a normal distribution with a mean of 0 and a variance of $\sigma_{g}^{2}$. The variance σ is adjusted adaptively according to Equation (25), where $\sigma_{0}$ is the initial variance, allowing for a gradual decrease in the mutation range as the algorithm progresses.

## 5. Experiment

We evaluate the proposed calibration method on the TUM infrastructure dataset and our own custom dataset. In this section, we describe the dataset and preparations, implementation detail, and qualitative and quantitative results analysis. For the qualitative performance evaluation, we compare the performance with Levinson’s static edge-based method [9] and Luo’s segmentation-based method [14], and compare the performance by the feature and optimization method. The experimental evaluation metric used was the root mean square error (RMSE). Static edge-based and proposed methods used 30 consecutive frames, and the segmentation-based method was conducted three times by accumulating 10 frames each according to the author’s recommendation.

### 5.1. Dataset and Preparations

#### 5.1.1. TUM Dataset

The TUM dataset [6] for traffic at busy intersections was acquired with synchronized data between cameras and LiDAR sensors. It contains 4800 images and point clouds. The dataset includes complex driving scenes at intersections, such as left turns, right turns, overtaking, and U-turns. The sensors of the TUM dataset consist of two Basler ace acA1920-50gc cameras (Basler AG, Ahrensburg, Germany) and Ouster OS1-64 LiDARs (Ouster, Inc., San Francisco, CA, USA). The camera–LiDAR extrinsic ground truth parameters were obtained by applying the method proposed in [8]. The TUM dataset was used to compare the accuracy with the static edge-based method and segmentation-based method.

#### 5.1.2. Own Dataset

Our own dataset contains data from a Korean intersection environment, with two CCTVs and one Hesai 40ch LiDAR installed in the infrastructure. A total of 30 min of traffic data was acquired from the camera and LiDAR. It is synchronized at a rate of 10 Hz. The ground truth extrinsic parameters were established by manually extracting distinct features such as poles, traffic signs, and building corners—from both camera and LiDAR data. These LiDAR features were projected onto the camera image plane, and the pixel distances between the projected LiDAR features and their corresponding camera features were measured. Extrinsic parameters were accepted as ground truth when the average pixel distance was within 1 pixel. The TUM and own datasets were used to compare the accuracy by edge features and optimization methods. Experiments were conducted using 30 frames from the TUM dataset and own dataset to compare each edge feature and optimization method.

### 5.2. Experiment Details

Table 2 outlines the hyperparameters employed in the feature extraction process of our camera–LiDAR calibration method. These parameters are categorized into five key areas: Ground Removal, DBSCAN Clustering, Morphological Operations, Dynamic Object Detection, and Adaptive Edge Feature Thresholding. Each category includes specific parameters, their assigned values, and corresponding descriptions. This detailed specification ensures the reproducibility of our experiments and facilitates the optimization of the calibration algorithm by clearly defining the settings used for each feature extraction stage.

Table 3 shows the hyperparameters used in the GA algorithm. Reproducibility is an important factor in experiments for genetic algorithms. We explain the detailed parameters for reproducibility in experiments. The population size starts the search through various initial solutions by generating 100 genes within the range of twice the calibration error. A selection pressure of 1.3 is used to maintain a balance between diversity and convergence. The crossover probability is 0.25, which indicates that the selected object pair has a 25% chance of undergoing crossover to generate offspring. This creates offspring individuals with new genes while maintaining the information of the parent genes. In addition, the mutation probability is set to 0.01 to randomly provide small mutations to maintain genetic diversity and prevent premature convergence. We initialize the Adaptive Blend Crossover parameter $\alpha_{0}$ and the Adaptive Mutation parameter $\sigma_{0}$ at 0.3. These parameters are crucial for dynamically adjusting crossover and mutation processes based on the current state of the generation. Finally, it is repeated for 100 generations so that the genetic algorithm can evolve toward estimating the optimal calibration parameters. These hyperparameter adjustments should be carefully determined as they affect the performance of the genetic algorithm.

### 5.3. Comparison Experiments

As shown in Table 4, we compared our method with the static edge-based method and the segmentation-based method. The static edge-based method enhanced the continuity of the objective function by applying the inverse distance transform to all edge features, including background features in the image, and extracted all depth-discontinuous edge features, including those from the background, in the point cloud. The segmentation-based method applied the segment anything model to the image and separated objects in the point cloud through clustering. To compare the calibration performance of each method, we generated extrinsic parameter errors. The error values were randomly sampled within the ranges of [1°, 0.1 m], [5°, 0.5 m], and [10°, 1.0 m].

The experimental results demonstrate that by utilizing a genetic algorithm and dynamic edge features, the proposed method achieves the highest calibration accuracy across a wide error range of [10°, 1.0 m]. The static edge-based method shows lower calibration accuracy due to its tendency to converge on local optima. This reduced performance results from extracting non-corresponding background edges, which influence noise in the calibration process and degrade accuracy. The proposed method addresses this limitation by selectively extracting only the dynamic edge features from both images and point clouds, effectively minimizing the impact of non-corresponding noise.

The segmentation-based method shows a significant decrease in performance over a wide search range. This is due to the algorithm’s characteristic of being influenced by the initial parameters, as projected LiDAR points within the segmentation mask region affect accuracy. Consequently, in cases of large initial errors, such as [10°, 1.0 m], accuracy decreases when fewer LiDAR points are projected within the mask region.

**Figure 6:**
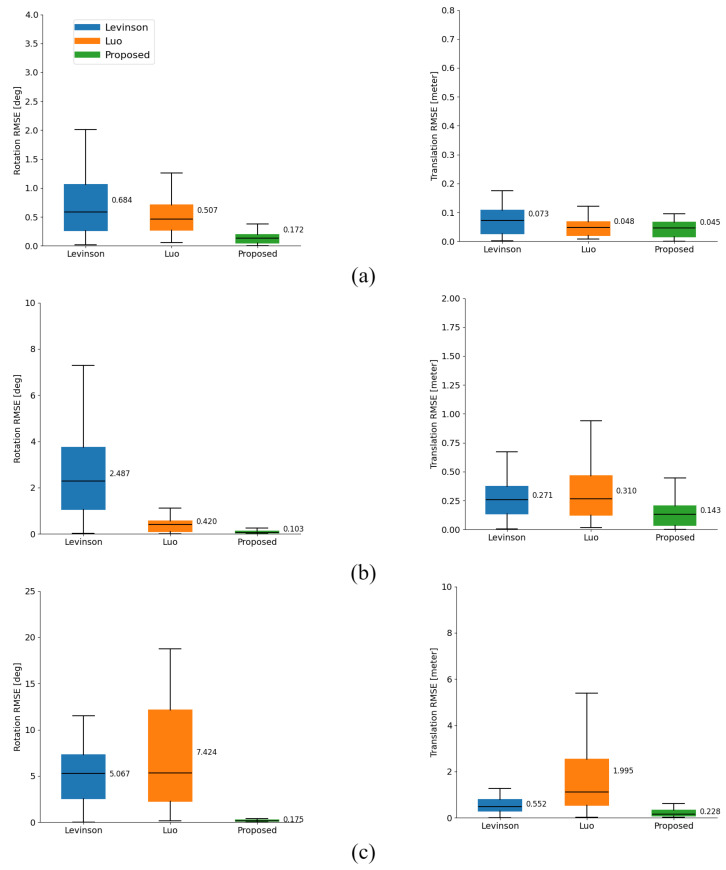
Comparison of accuracy by search range with existing edge-based and semantic-based methods. (**a**) Calibration RMSE median and deviation within [1°, 0.1 m]; (**b**) RMSE median and deviation within [5°, 0.5 m]; (**c**) RMSE median and deviation within [10°, 1.0 m].

**Figure 7:**
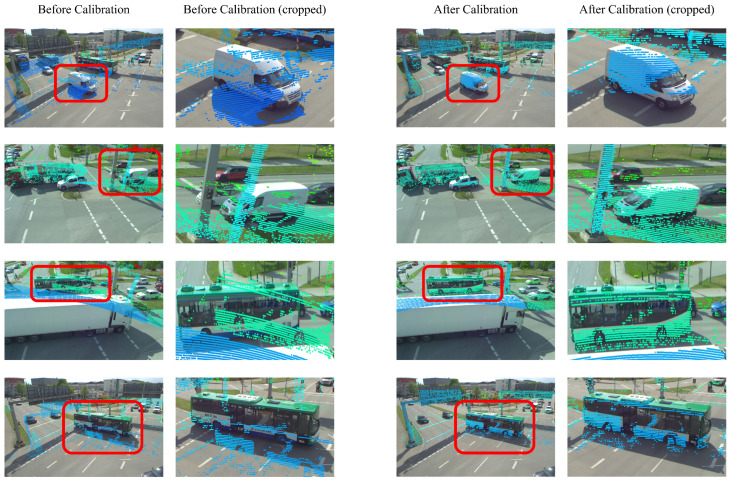
Proposed calibration qualitative results in TUM dataset. The first and second columns show the results before calibration. The third and fourth columns show the results after calibration.

Figure 6 shows the experimental results using 30 camera and LiDAR data pairs. Figure 6a is the result with a small error, and since Levinson’s method extracts a wide range of edges and performs calibration, we can see that the deviation is significant. In Figure 6b,c, we can see that the translation error of Luo’s method increases from within 0.5 m, and the rotation error does not diverge. Both methods show a significant deviation and an increase in the average error in a wide range. Figure 7 shows the qualitative results, which confirm that the proposed method converges to the ground truth even with the most significant error. Table 5 shows that the parameters estimated by the proposed method are close to the ground truth.

### 5.4. Calibration Results in Feature and Optimization Method

Table 6 compares calibration accuracy across two feature extraction methods, static edge and dynamic edge, using grid search and genetic algorithm optimizations, based on the TUM and own datasets. For static edge with grid search, the results show higher rotation errors, especially in the TUM dataset, with a mean rotation RMSE of 9.096° and a significant roll error of 16.661°. Similar trends are observed in the own dataset, where the mean rotation error reached 13.031°. The genetic algorithm significantly improved accuracy, reducing the mean rotation RMSE to 1.948° for the TUM dataset and further to 2.911° in the own dataset. Additionally, translation errors also decreased to 0.739 m and 0.527 m, respectively. The dynamic edge method, particularly when combined with the genetic algorithm, provided the best results, with the TUM dataset achieving a mean rotation RMSE of 0.523° and a translation RMSE of 0.022 m.

**Figure 8:**
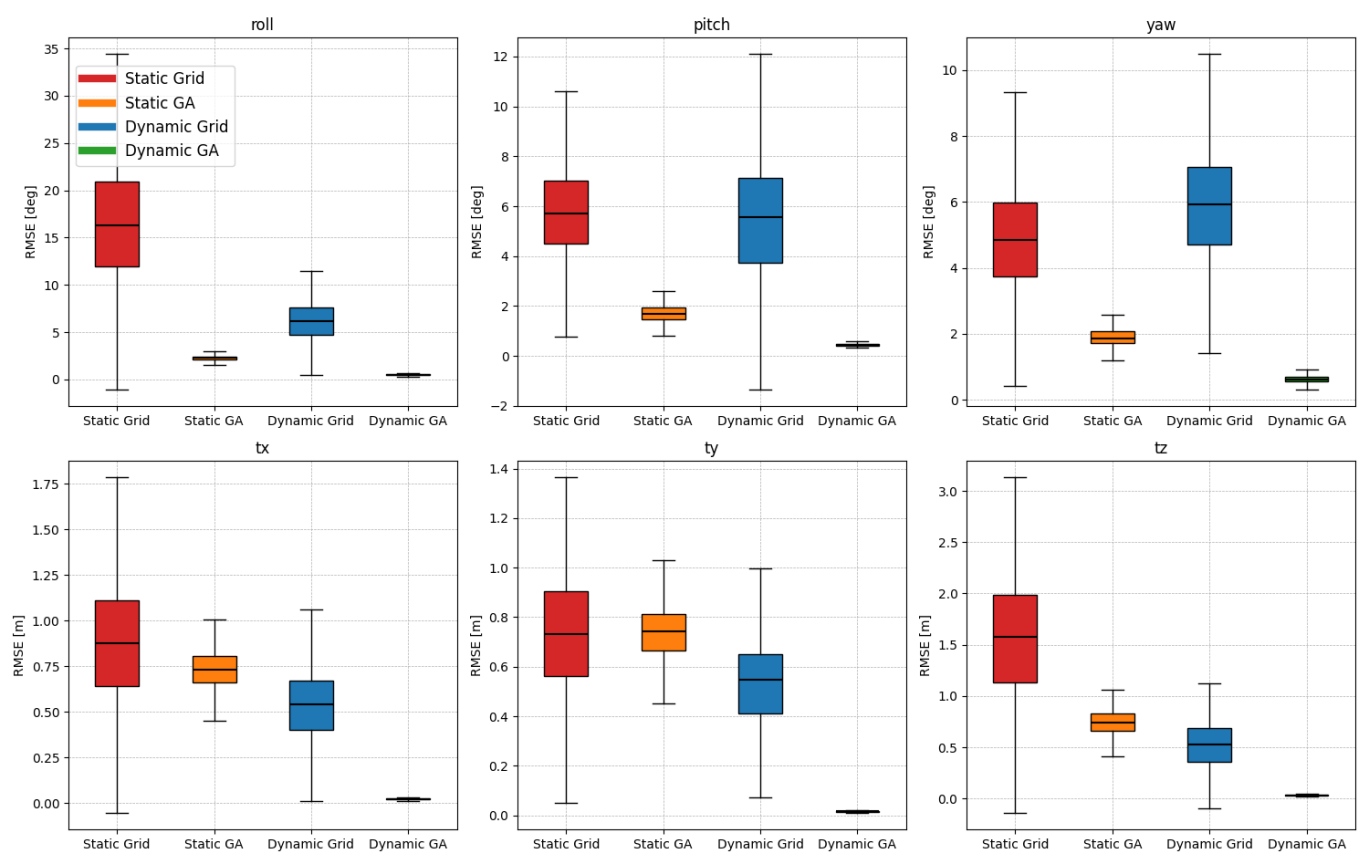
Comparison of RMSE values for rotation (roll, pitch, and yaw) and translation (tx, ty, and tz) parameters obtained from different feature and optimization methods (static grid search, static GA, dynamic grid search, and dynamic GA) using the TUM dataset. The box plots illustrate the distribution of RMSE values, emphasizing the calibration accuracy of each method.

**Figure 9:**
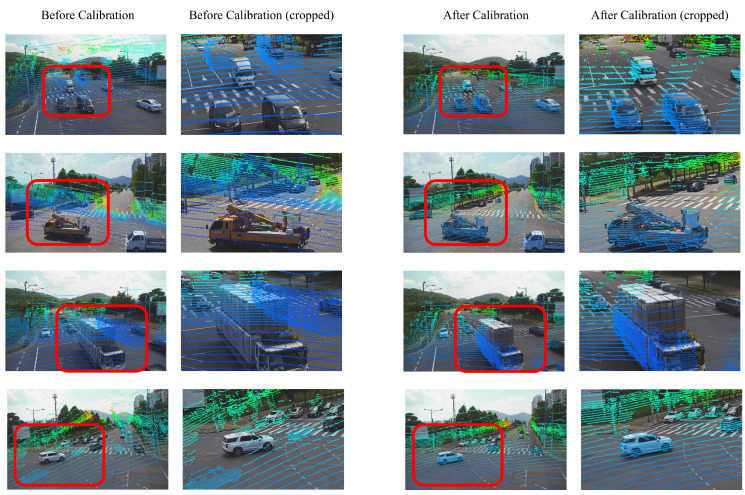
Proposed calibration qualitative results in own dataset. The first and second columns show the results before calibration. The third and fourth columns show the results after calibration.

The own dataset showed similar improvement with a mean rotation RMSE of 0.381°. Figure 8 shows the results of repeated experiments of optimization methods and feature extraction methods. We can see the effect of the optimization method on convergence. The grid search method shows a significant error in the results of repeated experiments. While the GA exhibits minor deviations, it demonstrates convergence to a local optimum when employing a static edge extraction method. Figure 9 shows precise alignment between the point cloud and image, indicating accurate visual matching. Additionally, the values in Table 7 confirm that the estimated parameters are numerically close to the ground truth, demonstrating the method’s reliability in achieving accurate calibration.

## 6. Conclusions

This study proposes a camera–LiDAR calibration method based on a real-valued genetic algorithm. This approach effectively filters out static background edge noise by utilizing the dynamic edge features of vehicles moving on the road, thereby performing calibration over a wide range. In particular, we achieve global optimal convergence of the calibration process by using dynamic object-based feature extraction despite the complex edge patterns in the road environment. In addition, we perform optimization on the genetic algorithm to verify its ability to avoid local optima and find a global optimal solution in repeated experiments. The experimental results are quantitatively verified, and the algorithm’s performance is demonstrated over a wide search range on both the TUM and our own datasets.

Future research will focus on further optimizing the proposed algorithm’s real-time processing capability and expanding its applicability in various environments. These advances are expected to enhance the method’s practical applicability in road traffic management systems, improve calibration accuracy, and ultimately, contribute to more efficient traffic control. 

## Figures and Tables

**Table 1:** Notation summary. Bold symbols (e.g., $P^{L}$) represent point cloud and sets for clarity in distinguishing different data types.

Symbol	Definition	Dimension
*R*	Rotation matrix for aligning camera and LiDAR coordinates	3×3
*T*	Translation vector between camera and LiDAR	3×1
*K*	Intrinsic matrix for LiDAR-to-image projection	3×3
$d_{f,i}$	Distance between nearest points in successive frames	Scalar
*D*	Depth discontinuity value calculated between two point clouds	Scalar
KCF(R,T)	Kernel correlation function for fitness evaluation	Scalar
Prob(rank)	Selection probability by rank in genetic algorithm	Scalar
gene	Genetic vector with six real-valued elements	$R^{6}$
$p_{n}^{I}$	Single projected LiDAR point in image plane coordinates	$Z^{2}$
$P^{L}$	LiDAR point cloud in LiDAR coordinates	$R^{3}$
$P^{C}$	LiDAR point cloud in camera coordinates	$R^{3}$
$P^{I}$	LiDAR point cloud in image coordinates	$Z^{2}$
${\mathrm{dyn}}^{L}$	Moving object detection result in LiDAR’s point cloud	$Z^{2}$
${\mathrm{dyn}}^{C}$	Moving object detection result in image pixels	$Z^{2}$
${\mathrm{edge}}^{L}$	3D dynamic object edge in image coordinates	$Z^{2}$
${\mathrm{edge}}^{C}$	2D dynamic object edge in image coordinates	$Z^{2}$
ch	Chromosome composed of gene	6

**Table 2:** Hyperparameters for feature extraction.

Parameter	Value	Description
Ground Removal		
Ground-plane fitting	RANSAC	Method to estimate and remove ground points
Ground threshold	0.35 m	Distance from ground plane for removing points
DBSCAN Clustering		
ε (epsilon)	0.7 m	Radius for neighborhood search
minCorePoints	15	Minimum points to form a cluster
Morphological Operations		
Kernel size	3×3	Structural element for dilation/erosion
Iterations	1	Number of dilation/erosion loops
Dynamic Object Detection		
$th_{\mathrm{dynamic}}^{L}$	0.1 m	Threshold for LiDAR dynamic object
$th_{\mathrm{dynamic}}^{C}$	30	Threshold for camera dynamic object
Adaptive Edge Feature Thresholding		
$th_{depth}^{\prime }$	Adaptive by distance	Adjusted based on object distance from LiDAR
$th_{low}^{\prime }$, $th_{high}^{\prime }$	Adaptive by mask area	Adjusted based on object mask size in camera image

**Table 3:** Hyperparameters for the real-coded genetic algorithm.

Hyperparameter	Value
Population size	100
Selection pressure	1.3
Crossover probability	0.25
Mutation probability	0.01
Adaptive Blend Crossover initial value ($\alpha_{0}$)	0.3
Adaptive Mutation initial value ($\sigma_{0}$)	0.3
Number of Generations	100

**Table 4:** Accuracy comparison according to calibration range in TUM dataset. (Bold values indicate the lowest errors).

Method	Feature	Optimization	Range	Rotation RMSE (°)	Translation RMSE (m)
Levinson et al. [9]	Static edge	Grid search	[1°, 0.1 m]	0.684	0.073
			[5°, 0.5 m]	2.487	0.271
			[10°, 1.0 m]	5.067	0.552
Luo et al. [14]	Segmentation	Grid search	[1°, 0.1 m]	0.507	0.048
			[5°, 0.5 m]	0.420	0.310
			[10°, 1.0 m]	7.424	1.995
Ours	Dynamic edge	Genetic algorithm	[1°, 0.1 m]	**0.172**	**0.045**
			[5°, 0.5 m]	**0.103**	**0.143**
			[10°, 1.0 m]	**0.175**	**0.228**

**Table 5:** Comparison of ground truth and estimated parameters from camera 1 of TUM dataset.

	Rotation (°)			Translation (m)		
	Roll	Pitch	Yaw	x	y	z
Ground truth	118.581	−47.160	−20.866	1.962	1.053	−1.301
Estimated value	118.432	−47.354	−21.040	1.713	0.842	−1.523
Error	0.149	0.194	0.174	0.249	0.211	0.222

**Table 6:** Comparison of calibration accuracy by feature and optimization method in own dataset. (Bold values indicate the lowest errors).

Feature	Optimization	Dataset	Rotation RMSE (°)				Translation RMSE (m)			
			Mean	Roll	Pitch	Yaw	Mean	X	Y	Z
Static edge	Grid search	TUM	9.096	16.661	5.712	4.916	0.874	0.729	0.346	1.547
		Own dataset	13.031	15.429	18.981	4.684	0.758	1.409	0.159	0.705
	Genetic algorithm	TUM	1.948	2.258	1.696	1.891	0.739	0.741	0.731	0.745
		Own dataset	2.911	2.756	3.149	2.829	0.527	0.529	0.525	0.527
Dynamic edge	Grid search	TUM	5.865	6.098	5.548	5.948	0.532	0.533	0.532	0.530
		Own Dataset	4.432	4.349	3.998	4.950	0.454	0.453	0.454	0.456
	Genetic algorithm	TUM	**0.523**	**0.507**	**0.442**	**0.620**	**0.022**	**0.016**	**0.018**	**0.032**
		Own dataset	**0.381**	**0.363**	**0.443**	**0.338**	**0.178**	**0.175**	**0.187**	**0.172**

**Table 7:** Comparison of ground truth and estimated parameters from camera 1 of own dataset.

	Rotation (°)			Translation (m)		
	Roll	Pitch	Yaw	x	y	z
Ground truth	−94.683	−17.752	−3.260	−5.252	4.414	0.443
Estimated value	−95.046	−18.195	−3.598	−5.430	4.227	0.271
Error	0.363	0.443	0.338	0.178	0.187	0.172

## Data Availability

Data will be made available on request.
